# Venoarterial Extracorporeal Membrane Oxygenation Implementation in Septic Shock Rat Model

**DOI:** 10.1097/MAT.0000000000002168

**Published:** 2024-02-29

**Authors:** Tianlong Wang, Mingru Zhang, Wenhao Dong, Jing Wang, Han Zhang, Yuefu Wang, Bingyang Ji

**Affiliations:** From the * Department of Cardiopulmonary Bypass, Fuwai Hospital, National Center for Cardiovascular Disease, State Key Laboratory of Cardiovascular Medicine, Chinese Academy of Medical Sciences & Peking Union Medical College, Beijing, China; † Department of Anesthesiology, Beijing Tongren Hospital, Capital Medical University, Beijing, China; ‡ Surgical IntensiveCare Unit, Beijing Shijitan Hospital, Capital Medical University, Beijing, China.

**Keywords:** septic shock, venoarterial extracorporeal membrane oxygenation, lipopolysaccharides, rat, animal model

## Abstract

Septic shock, a global health concern, boasts high mortality rates. Research exploring the efficacy of venoarterial extracorporeal membrane oxygenation (VA-ECMO) in septic shock remains limited. Our study aimed to establish a rodent model employing VA-ECMO in septic shock rats, assessing the therapeutic impact of VA-ECMO on septic shock. Nineteen Sprague–Dawley rats were randomly assigned to sham, septic shock, and (septic shock + VA-ECMO; SSE) groups. Septic shock was induced by intravenous lipopolysaccharides, confirmed by a mean arterial pressure drop to 25–30% of baseline. Rats in the SSE group received 2 hours of VA-ECMO support and 60 minutes of post-weaning ventilation. Sham and septic shock groups underwent mechanical ventilation for equivalent durations. Invasive mean arterial pressure monitoring, echocardiographic examinations, and blood gas analysis revealed the efficacy of VA-ECMO in restoring circulation and ensuring adequate tissue oxygenation in septic shock rats. Post-experiment pathology exhibited the potential of VA-ECMO in mitigating major organ injury. In summary, our study successfully established a stable septic shock rat model with the implementation of VA-ECMO, offering a valuable platform to explore molecular mechanisms underlying VA-ECMO’s impact on septic shock.

Sepsis is a global health burden and is responsible for 20% of annual global deaths.^1,2^ A substantial portion of sepsis patients progress to severe sepsis or septic shock, resulting in mortality rates of up to 40%.^3^ Among adult patients experiencing septic shock, approximately one-third to one-half also present with concurrent left ventricular dysfunction, commonly known as septic cardiomyopathy.^4–6^ These patients exhibit refractory circulatory failure and demonstrate a higher mortality rate than patients solely afflicted with septic shock.^7^ Fluid resuscitation and vasopressor administration are standard interventions to preserve hemodynamic stability in patients with septic shock, but their efficacy is diminished in septic shock patients with concurrent cardiac dysfunction.^8^ Currently, there are no effective life-saving strategies for these patients.

Venoarterial extracorporeal membrane oxygenation (VA-ECMO) is an increasingly employed method of circulatory assistance for patients experiencing refractory cardiac or cardiopulmonary failure.^9^ In VA-ECMO, venous blood is drained from the body, oxygenated in the membrane oxygenator, and returned to the arterial system, bypassing the heart and lungs to simultaneously provide respiratory and hemodynamic support.^10^ For septic shock patients with severe cardiac dysfunction, VA-ECMO may present as a potential option for life-supporting intervention.^8,11^ However, the efficacy of VA-ECMO in treating patients with septic shock remains uncertain. Therefore, we aim to establish a rodent VA-ECMO model in septic shock rats. This model could offer the potential for further investigation into molecular mechanisms on the effects of VA-ECMO in septic shock and provide more guiding evidence for clinical practice.

## Materials and Methods

### Animals

The whole protocol received review and approval from the Institutional Animal Care and Use Committee, Fuwai Hospital, Chinese Academy of Medical Sciences (FW-2023-0042). All experimental procedures complied with the Guide for the Care and Use of Laboratory Animals published by the National Institutes of Health. The whole study followed the animal research: reporting of in vivo experiments (ARRIVE) guidelines (http://www.nc3rs.org.uk/arrive).

The male Sprague–Dawley (SD) rats (350–450 gm) were kept under standard laboratory conditions, with free access to food and water (originally sourced from the HFK Bioscience, China). Nineteen SD rats were randomly assigned into three groups: sham group (n = 5), septic shock group (n = 7), and (septic shock + VA-ECMO; SSE) group (n = 7).

### Anesthesia and Mechanical Ventilation

The rats were initially anesthetized in a box with 5% isoflurane in oxygen, then maintained at 2% after tracheal intubation. Endotracheal intubation was undertaken with a 16G endotracheal tube. Ventilatory parameters included a 40% inhalation oxygen concentration, 70–75 breaths/minute, a tidal volume of 10 ml/kg, and an inhalation-exhalation ratio of 1:1. Body temperature was measured with rectal thermometry.

### Septic Shock Rat Model

The septic shock rat model was induced by intravenous injection of lipopolysaccharides (LPS) as previously described.^12,13^ The left femoral artery was cannulated for continuous monitoring of mean arterial blood pressure (MAP) and heart rate (HR). The *Escherichia coli* 055:B5 (L-2880; Sigma-Aldrich, MO; 15 mg/kg, 5 mg LPS dissolved in 1 ml 0.9% saline) was slowly injected into the rats through the femoral vein in 10 minutes. Septic shock was achieved when MAP decreased to 25–30% of the baseline value, which was about 1.5 hours after the injection of LPS. For the sham group, saline (0.9%, 3 ml/kg) was injected. After the establishment of the septic shock model, the rats in the SSE group were supported with VA-ECMO.

### Venoarterial Extracorporeal Membrane Oxygenation Circuit

**Figure 1.**
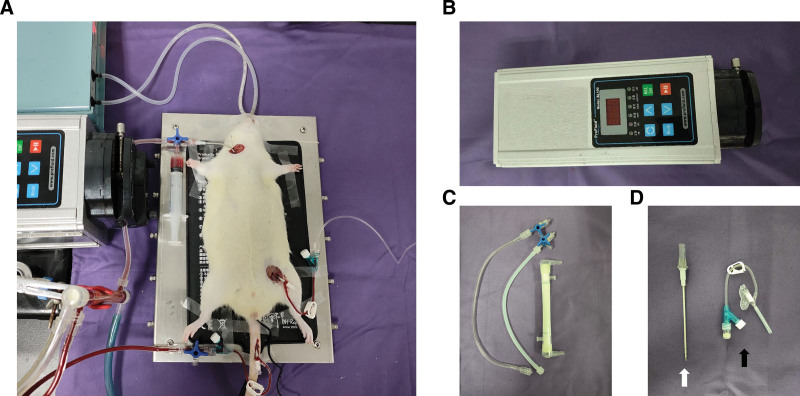
Rat VA-ECMO mode. **A**: The whole VA-ECMO circuit. **B**: Mini peristaltic pump. **C**: Membrane oxygenator and circuit tubing. **D**: White arrow, 16G specially designed catheter as venous drainage cannula. Black arrow, 22G arterial perfusion cannula. VA-ECMO, venoarterial extracorporeal membrane oxygenation.

In this study, the VA-ECMO rat model was modified according to the previous Cardiopulmonary bypass (CPB) rat model reported by our team.^14,15^ The actual VA-ECMO rat model is shown in Figure 1A. The VA-ECMO circuit system consisted of a roller pump (Prefluid, Changzhou, China) (Figure 1B), a small membrane oxygenator (Kewei, Dongguan, China) with custom-designed connecting tubes (Figure 1C), and intravascular catheters (Figure 1D). The total circuit volume was about 6 ml consisting of 6% hydroxyethyl starch (4 ml) and saline with 100 U heparin (2 ml). First, a 22-gauge catheter was inserted into the tail artery as the arterial perfusion end of the VA-ECMO. Subsequently, a specially designed venous drainage tube was inserted in the right jugular vein as the venous drainage end of VA-ECMO. Heparin (250 U) was administered *via* the right jugular vein before the initiation of VA-ECMO. After systemic heparinization, VA-ECMO was initiated and the flow rate was increased stepwise up to 80–90 ml/kg/minute. The inspired oxygen concentration for VA-ECMO was maintained at 80%. Mean arterial blood pressure, HR, and rectal temperature were recorded throughout the process. After 2 hours support, the rats were weaned off the VA-ECMO, and the remaining priming volume was collected and reinfused. Then the rats were ventilated for another 60 minutes without VA-ECMO support. Postoperative ventilatory parameters were identical with the preoperative ones. For sham and septic shock groups, the rats only underwent cannulation and heparinized. At the end of the whole experiment, all rats were sacrificed and vital organs were collected for further histology analysis.

### Arterial Blood Gas and Biochemical Analyses

**Figure 2.**
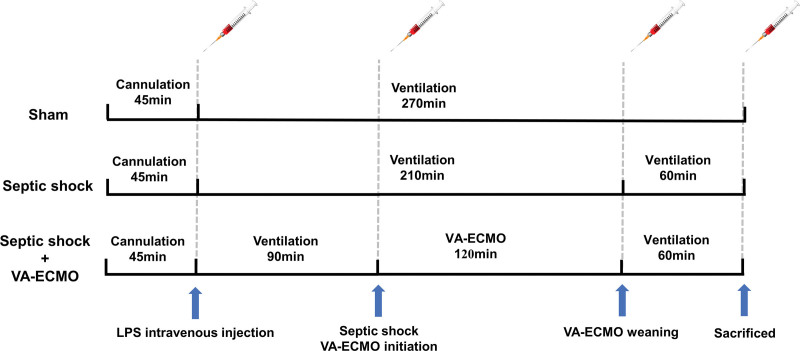
The experimental protocol and time points for blood gas measurement. LPS, lipopolysaccharides; VA-ECMO, venoarterial extracorporeal membrane oxygenation.

During the whole experiment, 0.5 ml arterial blood was obtained *via* the left femoral artery at each of the four predefined time points and immediately taken for blood gas analysis. The measurements were performed using the i-STAT portable clinical blood gas analyzer (Abbott Laboratories, Abbott Park, IL). The whole experimental protocol and predefined time points for blood gas and biochemical parameters measurement are depicted in Figure 2.

### Echocardiographic Examinations

Echocardiography was performed to evaluate cardiac function at three predefined time points (before surgery; VA-ECMO initiation, sacrificed). The Vinno 6th ultrasound imaging system (Vinno Corporation, Suzhou, China) was used for echocardiography measurements. All measurements were done three times, averaged, and analyzed by experienced cardiologists who were blinded to the experiment protocol.

### Histology

To evaluate the pathologic findings of vital organs in each group, the heart, liver, hippocampal, ileum, and kidney tissue samples were fixed in 4% formaldehyde, embedded with paraffin, and divided into 4 µm sections for hematoxylin-eosin (HE) staining. The hepatic, hippocampal, intestinal, and renal pathologic scores were graded according to the methods described previously.^16–19^ The scoring rules are shown in Supplementary Table 1, Supplemental Digital Content, http://links.lww.com/ASAIO/B224.

### Statistics

All data are presented as the mean ± standard deviation. Graphs were drawn using GraphPad Prism 9.0 software (GraphPad, San Diego, CA). Student’s t-tests were used for comparisons between the two groups. *p* < 0.05 were considered to be statistically significant.

## Results

### Experimental Overview

**Figure 3.**
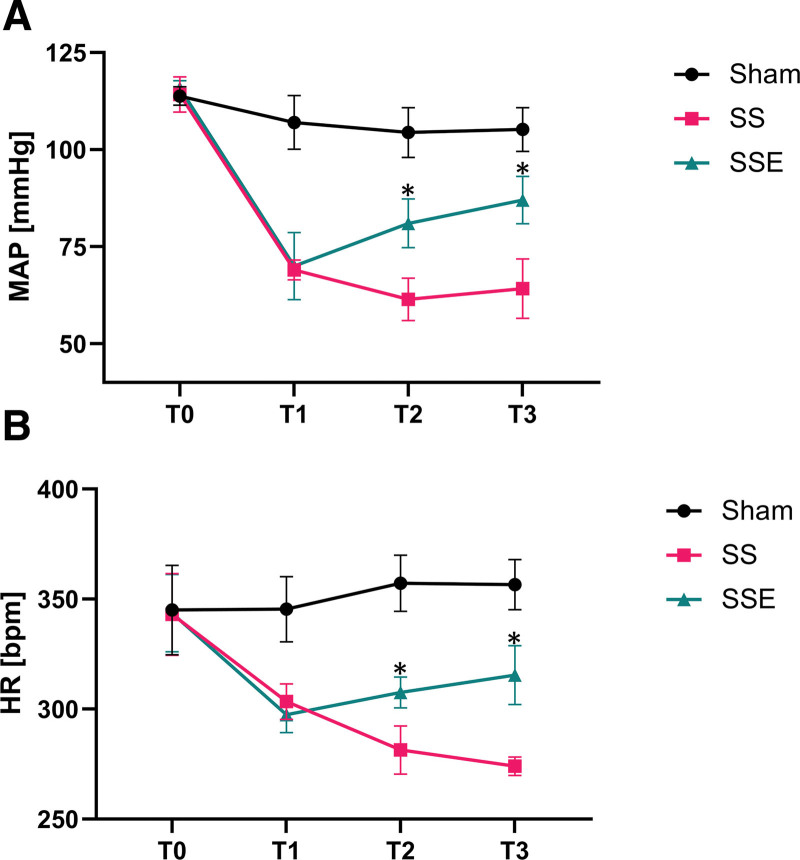
Time course of changes in hemodynamic parameters. **A, B**: MAP and HR were measured over time in the three groups (values: mean ± standard error). *p < 0.05 *vs.* sepsis shock group at the same time points. bpm, beat per minute; HR, heart rate; MAP, mean arterial pressure; SS, septic shock; SSE, septic shock + VA-ECMO; T0, baseline; T1, septic shock and VA-ECMO initiation; T2, VA-ECMO weaning; T3, sacrificed; VA-ECMO, venoarterial extracorporeal membrane oxygenation.

All rats were survived to the experimental end-point. The MAP and HR data were collected at T0 (Baseline), T1 (Septic shock and VA-ECMO initiation), T2, (VA-ECMO weaning), and T3 (Sacrificed) and presented in Figure 3. Septic shock was defined as once the rats’ MAP decrease was about 25–30% of the baseline value, which occurred about 1.5 hours after the LPS injection and continued to fall below 50% at approximately 4 hours after the injection. However, after applying VA-ECMO, real-time blood pressure monitoring showed that rats in the SSE group performed higher MAP and more standard blood pressure waveforms than the septic shock group (Supplementary Figure 1, Supplemental Digital Content, http://links.lww.com/ASAIO/B222). Furthermore, the increasing trend of MAP was still in place after VA-ECMO weaning (Figure 3A). Heart rate data revealed the same trend as MAP in each group (Figure 3B). These data indicate a successful rat model of septic shock, characterized by progressively deteriorating hemodynamic states following intravenous injection of LPS. However, the administration of VA-ECMO treatment resulted in a notable recovery of these hemodynamic parameters.

### Blood Gas and Biochemical Test Results

The blood gas and biochemical parameters are summarized in Table 1. The levels of hemoglobin and hematocrit were lower after VA-ECMO initiation but the PaO_2_ was maintained above normal values. However, after the withdrawal of the VA-ECMO, PaO_2_ was difficult to maintain in normal. In addition, both septic shock and SSE groups showed continued elevation of lactate, but no differences were found between the two groups at each time point. It should be noticed that the serum creatinine and blood urea nitrogen levels of rats in SSE group were reduced after receiving VA-ECMO treatment compared with septic shock group.

**Table 1.** Blood Gas and Biochemical Test Parameters of the Rats

	Group	T0	T1	T2	T3
Hb (g/L)	Sham	13.1 ± 0.8	12.6 ± 0.9	13.2 ± 0.8	13.5 ± 0.9
	Septic shock	12.9 ± 0.7	13.6 ± 1.1	13.2 ± 1.2	12.6 ± 1.0
	Septic shock + VA-ECMO	13.6 ± 0.8	12.6 ± 0.9	9.6 ± 0.7*†	11.6 ± 1.2†
Hct (%)	Sham	38 ± 2.4	37 ± 2.8	39 ± 2.3	40 ± 2.5
	Septic shock	38 ± 1.9	40 ± 3.1	39 ± 3.5	37 ± 3.0
	Septic shock + VA-ECMO	40 ± 2.3	37 ± 2.6	28 ± 2.1*†	34 ± 3.6*†
pH	Sham	7.41 ± 0.06	7.39 ± 0.05	7.40 ± 0.03	7.40 ± 0.04
	Septic shock	7.43 ± 0.06	7.40 ± 0.02	7.33 ± 0.09	7.25 ± 0.07*
	Septic shock + VA-ECMO	7.43 ± 0.02	7.42 ± 0.02	7.44 ± 0.11	7.38 ± 0.04†
PaO_2_ (mm/Hg)	Sham	187 ± 20	181 ± 23	191 ± 13	173 ± 9
	Septic shock	181 ± 11	135 ± 8*	116 ± 6*	117 ± 12*
	Septic shock + VA-ECMO	183 ± 14	138 ± 9*	263 ± 17*†	152 ± 10*†
PaCO_2_ (mm/Hg)	Sham	45 ± 3	45 ± 3	43 ± 4	40 ± 3
	Septic shock	43 ± 3	44 ± 2	40 ± 6	37 ± 5
	Septic shock + VA-ECMO	43 ± 5	43 ± 4	38 ± 5	33 ± 4*
Lac (mmol/L)	Sham	0.53 ± 0.23	1.15 ± 0.38	1.51 ± 0.72	1.90 ± 0.63
	Septic shock	0.72 ± 0.33	1.80 ± 0.57	2.65 ± 1.12	4.76 ± 1.77*
	Septic shock + VA-ECMO	0.80 ± 0.41	1.74 ± 0.84	2.68 ± 0.85	4.18 ± 1.35*
HCO_3_− (mmol/L)	Sham	28.5 ± 3.5	27.7 ± 5.6	27.9 ± 2.5	29.8 ± 2.6
	Septic shock	29.3 ± 2.2	28.6 ± 2.8	20.7 ± 2.2*	19.3 ± 4.0*
	Septic shock + VA-ECMO	29.8 ± 3.4	27.9 ± 2.4	22.6 ± 3.4*	18.9 ± 2.7*
Na+ (mmol/L)	Sham	141 ± 3	140 ± 2	139 ± 1	138 ± 2
	Septic shock	142 ± 3	139 ± 4	141 ± 4	141 ± 3
	Septic shock + VA-ECMO	141 ± 4	141 ± 3	143 ± 4	138 ± 1
K+ (mmol/L)	Sham	3.5 ± 0.4	4.0 ± 0.3	3.7 ± 0.2	4.0 ± 0.4
	Septic shock	3.9 ± 0.4	4.1 ± 0.2	3.9 ± 0.3	4.0 ± 0.3
	Septic shock + VA-ECMO	3.5 ± 0.4	3.6 ± 0.5	4.0 ± 0.7	4.1 ± 0.6
Cl− (mmol/L)	Sham	104 ± 4	102 ± 3	104 ± 5	103 ± 4
	Septic shock	105 ± 3	106 ± 3	107 ± 4	106 ± 3
	Septic shock + VA-ECMO	103 ± 3	104 ± 3	103 ± 4	104 ± 2
BUN (mmol/L)	Sham	11.8 ± 2.3	13.8 ± 1.6	13.6 ± 2.3	15.2 ± 2.0
	Septic shock	11.2 ± 3.1	17.4 ± 2.3	28.0 ± 4.7*	39.0 ± 2.9*
	Septic shock + VA-ECMO	11.0 ± 3.2	16.0 ± 4.1	24.2 ± 3.4*	30.0 ± 4.3*†
Cr (mmol/L)	Sham	0.2 ± 0.0	0.2 ± 0.0	0.3 ± 0.1	0.3 ± 0.1
	Septic shock	0.2 ± 0.0	0.3 ± 0.1	0.7 ± 0.1*	1.0 ± 0.2*
	Septic shock + VA-ECMO	0.2 ± 0.0	0.3 ± 0.1	0.4 ± 0.1*	0.6 ± 0.2*†

Variables are expressed by mean ± standard deviation.

* *p* < 0.05 *vs.* sham group at the same time points.

† *p* < 0.05 *vs.* sepsis shock group at the same time points.

BUN, blood urea nitrogen; Cr, creatine; Hb, hemoglobin; Hct, hematocrit; Lac, lactate; Lac, lactate; PaCO_2_, partial pressure of arterial carbon dioxide; PaO_2_, partial pressure of arterial oxygen; T0, baseline; T1, septic shock and VA-ECMO initiation; T2, VA-ECMO weaning; T3, sacrificed; VA-ECMO, venoarterial extracorporeal membrane oxygenation.

### Cardiac Function Evaluation

**Figure 4.**
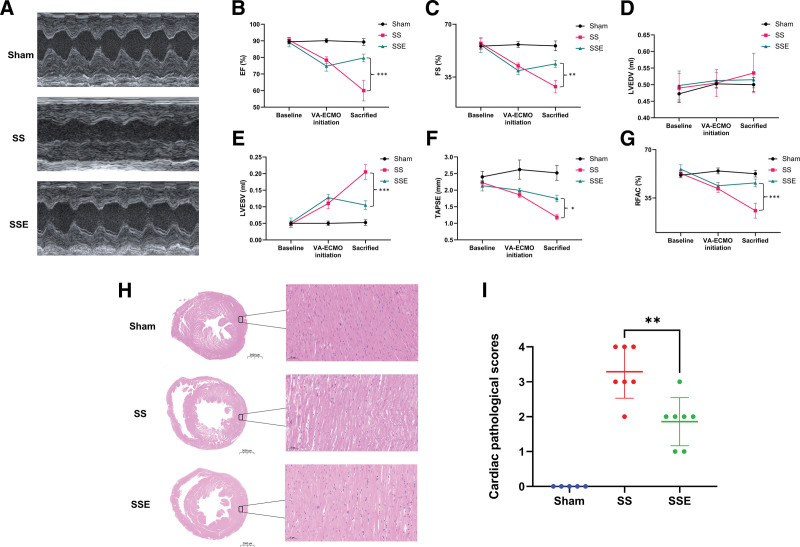
Cardiac function evaluation. **A**: Representative images of M-mode echocardiography in different groups. **B–G**: EF, FS, EDV, ESV, TAPSE, and RVFAC were recorded at three predefined time points (before surgery; VA-ECMO initiation, sacrificed) in three groups (n = 4 each group). **H, I**: Representative hematoxylin and eosin staining images of heart tissues for each group and graphical presentation of the cardiac pathologic scores. The histological analysis revealed enlarged cardiac chambers and thinner ventricular walls in the septic shock group. Under higher magnification, disordered muscle fibers, increased cell gap, and many broken fibers were found. Treatment with ECMO could ameliorate myocardial injury. Values: mean ± SEM; *p < 0.05, **p < 0.01, ***p < 0.001. EF, ejection fraction; FS, fractional shortening; LVEDV, left ventricular end-diastolic volume; LVESV, left ventricular end-systolic volume; RVFAC, right ventricular fractional area change; SS, septic shock; SSE, septic shock + VA-ECMO; TAPSE, tricuspid annular plane systolic excursion; VA-ECMO, venoarterial extracorporeal membrane oxygenation.

In the septic shock group, the administration of LPS *via* intravenous injection resulted in a rapid and progressive decline in left cardiac function, as evidenced by a decrease in both the ejection fraction (EF) and fractional shortening (FS), as well as an increase in left ventricular end-systolic volume (LVESV). However, there was no significant change observed in left ventricular end-diastolic volume (LVEDV) (Figure 4, A–E). Additionally, there was a notable reduction in right cardiac function in the septic shock group, as indicated by a gradual decrease in tricuspid annular plane systolic excursion (TAPSE) and right ventricular fractional area change (RVFAC) (Figure 4, F and G). The VA-ECMO treatment demonstrated the potential to reverse the decline in cardiac function, as indicated by the higher values of EF, FS, TAPSE, and RVFAC, and lower values of ESV compared to the septic shock group in the final stage of the experiment (Figure 4, A–G). The representative two-dimensional echocardiography images from an apical four-chamber view (displayed in Graphics Interchange Format) visually depicted the beneficial impact of VA-ECMO on the restoration of cardiac function in septic shock rats (Supplementary Figure 2, Supplemental Digital Content, http://links.lww.com/ASAIO/B223). Additionally, the postoperative pathologic examination revealed that VA-ECMO treatment could mitigate myocardial injury in septic shock rats (Figure 4, H and I).

### Histological Examination

**Figure 5.**
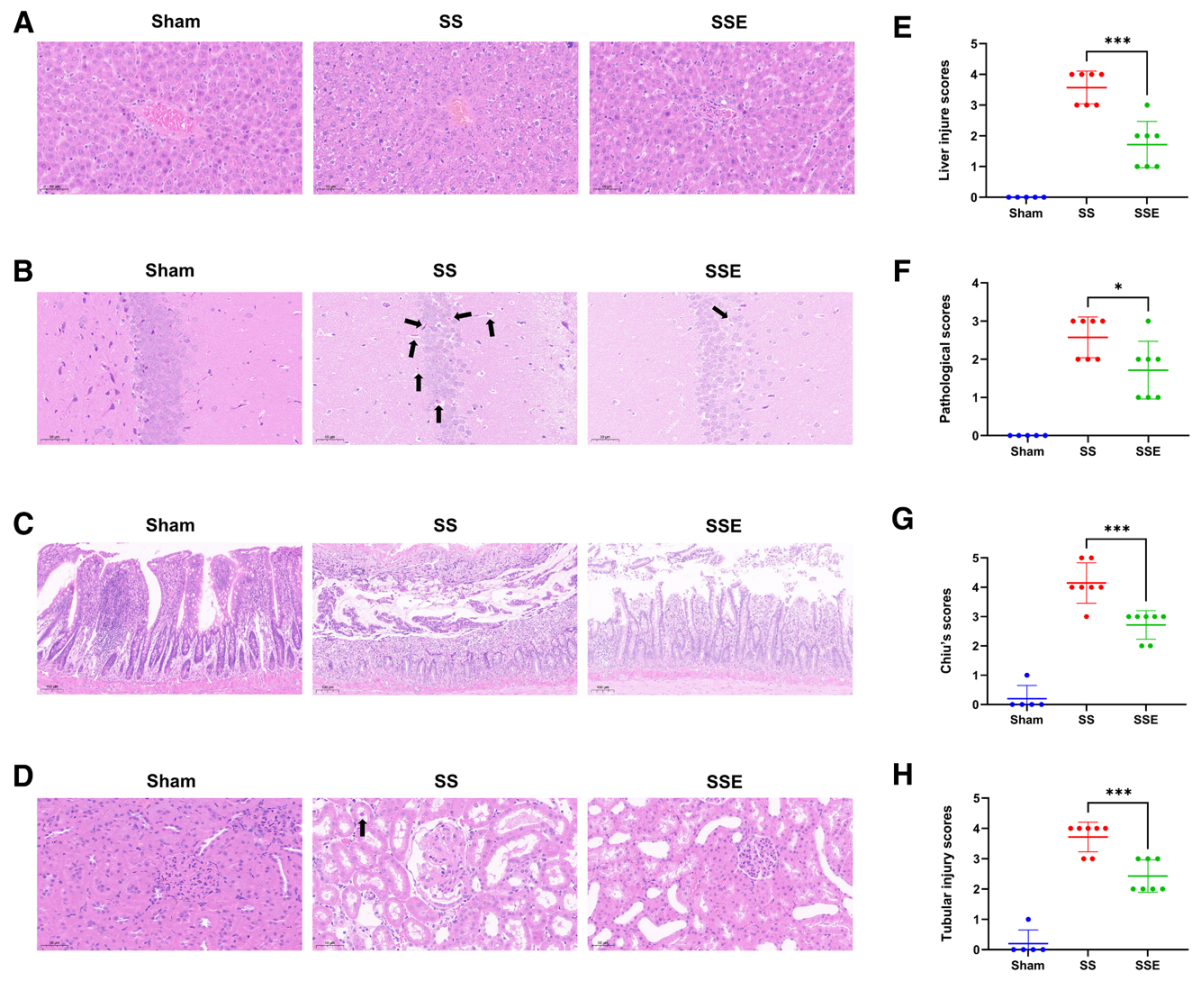
Hematoxylin and eosin staining assessed pathologic damage in the main organs. **A**, Representative liver micrographs of each group. The structure of liver lobules was severely damaged in the septic shock group. The use of VA-ECMO can maintain the structure of the liver lobule basically intact. **B**, Representative hippocampal CA3 region micrographs of each group. The number of hippocampal neurons decreased in the septic shock group and dark-stained and deformed neurons were identified (black arrows). VA-ECMO can alleviate neuronal damage induced by septic shock. **C**, Representative ileum micrographs of each group. In the septic shock group, the small intestine villi have fallen off. Treatment with ECMO could protect the basic integrity of the small intestinal villus. **D**, Representative kidney micrographs of each group. Tubular lumen enlargement, tubular formation, and shedding of epithelial cells (black arrow) were observed in the septic shock group. After applying VA-ECMO, tubular formation decreased and tubule injury improved. **E**, Graphical presentation of the liver injury scores. **F**, Graphical presentation of the pathologic scores. **G**, Graphical presentation of the Chiu’s scores. **H**, Graphical presentation of the renal tubular injury scores. Values: mean ± SEM; *p < 0.05, ***p < 0.001. SS, septic shock; SSE, septic shock + VA-ECMO; VA-ECMO, venoarterial extracorporeal membrane oxygenation.

Histological examination demonstrated that the rats in the septic shock group had severe lesions and extensive damage in the liver, hippocampus, intestinal, and kidney tissues. However, treating septic shock rats with VA-ECMO can attenuate tissue damage in main organs (Figure 5, A–D). Histopathological scoring of tissue damage showed, compared with the septic shock group, the SSE group had significantly decreased liver injury scores (Figure 5E; 1.71 ± 0.76 *vs*. 3.57 ± 0.53, *p* < 0.001), hippocampal pathologic scores (Figure 5F; 1.57 ± 0.53 *vs*. 2.43 ± 0.79, *p* = 0.030), Chiu’s scores of the ileum (Figure 5G; 2.71 ± 0.49 *vs*. 4.14 ± 0.69, *p* < 0.001) and tubular injury scores (Figure 5H; 2.43 ± 0.53 *vs*. 3.71 ± 0.49, *p* < 0.001).

## Discussion

At present, there is no specific treatment for septic shock. Because providing circulatory support, VA-ECMO is considered an optional life-saving method for patients with end-stage septic shock. In this study, we first established an economical, reliable, and reproducible VA-ECMO animal model in septic shock rats and preliminarily explored the therapeutic effect of VA-ECMO on septic shock with this model.

As a result of the positive outcomes of VA-ECMO in pediatric septic shock, it is recommended in some societal guidelines as a viable therapy.^20–22^ However, the application of VA-ECMO in adult septic shock remains controversial due to the following reasons. First of all, it should be noticed that different age groups exhibit different hemodynamic patterns in septic shock. The newborn is lack of complete closure of the ductus arteriosus. Hypoxia and acidosis caused by sepsis could increase pulmonary vascular resistance through the ductus arteriosus, resulting in persistent pulmonary hypertension and right heart failure. For young children with septic shock, low cardiac output and reduction in oxygen delivery caused by left heart failure are the main features. Conversely, the predominant cause of adult septic shock is vasomotor paralysis, and typically present with distributed shock.^23,24^ The main function of VA-ECMO is to partially or completely replace the heart function to maintain circulatory perfusion and is typically used to provide hemodynamic stabilization in the context of refractory cardiogenic shock but less frequently used for distributive shock.^25^ Other concerns including the introduction of foreign bodies into the vasculature during intravascular catheterization might contribute to the persistence of bacteremia, the contact between blood and foreign surfaces can exacerbate the systemic inflammatory response, and the coagulation dysfunction of septic patients would further predispose them to bleeding complications associated with ECMO.^26–29^ Nevertheless, existing clinical evidence does not entirely refute the potential significance of VA-ECMO in adult patients afflicted with end-stage septic shock. Recent studies indicate that the utilization of VA-ECMO may be a viable approach to yield survival advantages for septic shock patients experiencing severe cardiac dysfunction, particularly when conventional therapeutic interventions have proven ineffective.^8,30,31^ However, additional investigation into specific molecular mechanisms is warranted to substantiate and reinforce this perspective.

In this study, we chose an economical and rapid method to induce septic shock, that is, intravenous administration of a loading dose of LPS. A unique advantage of this septic model is that it can lead to a rapid decline of cardiac function in a short period. Significant reductions in major cardiac contractile echocardiogram parameters, including EF and FS for the left ventricle, and TAPSE and RVFAC for the right ventricle, were observed in septic shock rats. Additionally, postoperative cardiac pathology analysis clearly indicated myocardial damage in these rats. It is interesting that LPS-treated rats exhibited a significant decrease in LVESV but did not show a significant change in LVEDV. A previous study also reported that intravenous injection of LPS in rats did not alter LV end-diastolic pressure.^32^ Therefore, the overall manifestation of septic cardiomyopathy induced by intravenous injection of LPS in rats is an acute decrease in cardiac systolic function. Clinically, septic cardiomyopathy is characterized by acute myocardial depression with heart systolic dysfunction, similar to our septic shock rat model.^33^ Therefore, our septic shock rat model can appropriately translate clinical experience and simulate the application scenario of VA-ECMO advocated by clinical evidence.

After the intravenous administration of LPS to induce septic shock in rats, we proceeded to administer VA-ECMO for 2 hours. In clinical settings, the duration of VA-ECMO support tends to be longer than that of our model. To develop a more accurate VA-ECMO model that closely resembles the human condition, it is necessary to extend the duration of VA-ECMO runs. However, the current rat-adapted ECMO oxygenator and connecting tubes could not fully replicate existing clinical ECMO equipment, and prolonged extracorporeal circulation duration in rats may result in increased risks of systemic inflammatory reactions and complications. Previous research has demonstrated that the administration of extracorporeal circulation for 30 minutes in healthy adult rats could result in a notable elevation of inflammatory factors. Furthermore, extending extracorporeal circulation beyond 1 hour was associated with the emergence of various complications, including acute kidney injury.^34,35^ Therefore, when exploring the therapeutic effect of ECMO on rat disease models, a long duration of ECMO will aggravate the existing disease state. When referring to other ECMO rat models, most studies limit the duration of ECMO in rat models to 2 hours.^36,37^ Additionally, previous research and our pilot study both demonstrated that the septic shock rat model exhibits a considerable short-term lethality rate,^38^ thus the long experimental time will result in a significant amount of missing experiment data. Overall, maintaining VA-ECMO support for 2 hours and delaying observation for 1 hour after weaning can maximize the evaluation of whether VA-ECMO can produce a therapeutic effect in the rat septic shock model.

Blood gas and hemodynamic analyses revealed that VA-ECMO treatment effectively supplied oxygen to the tissues and maintained circulatory stability. Additionally, we noticed that VA-ECMO treatment effectively maintained the cardiac function of rats and avoided further deterioration of sepsis-related myocardial damage. This is consistent with current clinical evidence. Recent large-scale cohort study and meta-analyses have emphasized that ECMO treatment can be beneficial to patient prognosis for patients with septic shock accompanied by septic cardiomyopathy.^30,31^

Furthermore, subsequent pathologic analysis of the principal organs demonstrated that the implementation of VA-ECMO could alleviate tissue damage. This finding substantiates the point that the protective effects of VA-ECMO on septic shock rats persist even after ECMO weaning.

However, we must admit that this protective effect is limited, after the cessation of VA-ECMO support, it is still difficult for septic shock rats to maintain adequate oxygen supply through their intrinsic circulation. Furthermore, the tissue damage was still serious despite the mitigation observed. But at least, our study preliminary revealed that VA-ECMO may have a beneficial impact on mitigating the progression of severe septic shock. This finding was valuable as it implied that in future clinical practice when confronted with septic shock patients experiencing a severe reduction in cardiac function, early implementation of VA-ECMO could potentially delay the progression of septic shock and provide additional time for to make more efficient clinical decisions.

This study has the following limitations. 1) This experiment did not establish a positive control group consisting of septic shock rats treated with standard therapies. In this study, we aim to construct a stable septic shock rat model with decreased cardiac function and explore the therapeutic effect of VA-ECMO. Establishing a positive control group is not essential for substantiating the objectives of this study. In addition, the choice of standard therapy is challenging. Standard interventions for septic shock patients, such as fluid resuscitation and vasopressor, have limited efficacy in severe septic shock patients, especially with septic cardiomyopathy. Therefore, it differs from other ECMO applications where there is an appropriate treatment as a control, for example, CPR is a standard control treatment strategy for extracorporeal cardiopulmonary resuscitation (ECPR). Therefore, the selection of suitable standard positive control may mainly depend on how VA-ECMO improves the function of vital organs in septic shock patients. 2) Although the septic shock rat model in this study was facile, less time-consuming, and cost-effective, it is still different from the actual clinical practice. In clinical settings, septic shock usually arises from primary lesions, a gradual process that is not accounted for in this model. However, this septic shock rat model can induce stable circulatory failure and is suitable for the evaluation of the molecular mechanism underlying the efficacy of VA-ECMO in the treatment of septic. 3) This study did not assess the variations in inflammatory response between different groups, which warrants further investigation. A significant factor contributing to the limited acceptance of VA-ECMO implementation in septic shock is that blood contact with nonbiological surfaces would trigger the activation of the inflammatory response, thus leading to further deterioration of septic shock.

## Conclusions

In this study, the VA-ECMO model was first established in septic shock rats. Initial examination of physiologic parameters and histological analysis of major organs provided support evidence that VA-ECMO effectively mitigated circulatory collapse and minimized tissue injury in rats experiencing severe septic shock. The model established in this study serves as a foundation for future investigations into the molecular mechanisms underlying the tissue-protective effects of VA-ECMO in septic shock.

## Acknowledgments

The authors indebted to Dr. Gang Liu for experimental/equipment support.

## Supplementary Material


