# Supplementary material for: Venoarterial Extracorporeal Membrane Oxygenation Implementation in Septic Shock Rat Model
Source: ASAIO J. 2024 Feb 29;70(8):653–60. doi: 10.1097/MAT.0000000000002168 (PMC11280450; doi:10.1097/MAT.0000000000002168)
Supplement: Supplementary file 1 [file mat-70-0653-s001.pdf]

Supplementary Table 1.
Histology damage scoring rules.

**Argan**	**The details of scoring rules**
Liver	Liver injury scores:<br>0: no evident injury;<br>1: single-cell necrosis, minimal congestion or vacuolization;<br>2: necrosis <30%, mild<br>3: necrosis <60%, moderate congestion or vacuolization;<br>4: necrosis >60%, severe congestion or vacuolization
Hippocampus	Hippocampal pathological scores:<br>1: <20% affected area, necrotic neurons only in the most lateral areas;<br>2: 50% affected area, patchy areas in all sectors;<br>3: 75% affected area, with more extensive areas of necrotic neurons;<br>4: 100% affected, complete infarction of hippocampus including gyrus dentatus
Ileum	Chiu's scores:<br>1: Normal mucosal villi.<br>2: Development of subepithelial Gruenhagen's space, usually at the apex of the villus; often with capillary congestion.<br>3: Extension of the subepithelial space with moderate lifting of epithelial layer from the lamina propria.<br>4: Massive epithelial lifting down the sides of villi. A few tips may be denuded.<br>5: Denuded villi with lamina propria and dilated capillaries exposed. Increased cellularity of lamina propria may be noted.<br>6: Digestion and disintegration of lamina propria; hemorrhage and ulceration.
Kidney	Tubular injury scores:<br>0: no damage;<br>1: less than 25% damage;<br>2: 25%–50% damage;

	3: 50%–75% damage;<br>4: more than 75% damage.<br>Tubular damage is defined as loss of brush border, tubular dilation, cast formation, and cell lysis.
Heart	Cardiac pathological scores:<br>0: no abnormality<br>1: interstitial edema<br>2: cardiomyocyte edema, most of the myocardial fibers arranged regularly<br>3: cardiomyocytes arranged disorderly, with occasional red blood cell extravasation and inflammatory cell infiltration<br>4: myocardial fibers disorganized and ruptured, with inflammatory cell infiltration
