# Supplementary material for: Venoarterial Extracorporeal Membrane Oxygenation Implementation in Septic Shock Rat Model
Source: ASAIO J. 2024 Feb 29;70(8):653–60. doi: 10.1097/MAT.0000000000002168 (PMC11280450; doi:10.1097/MAT.0000000000002168)
Supplement: Supplementary file 2 [file mat-70-0653-s002.pdf]

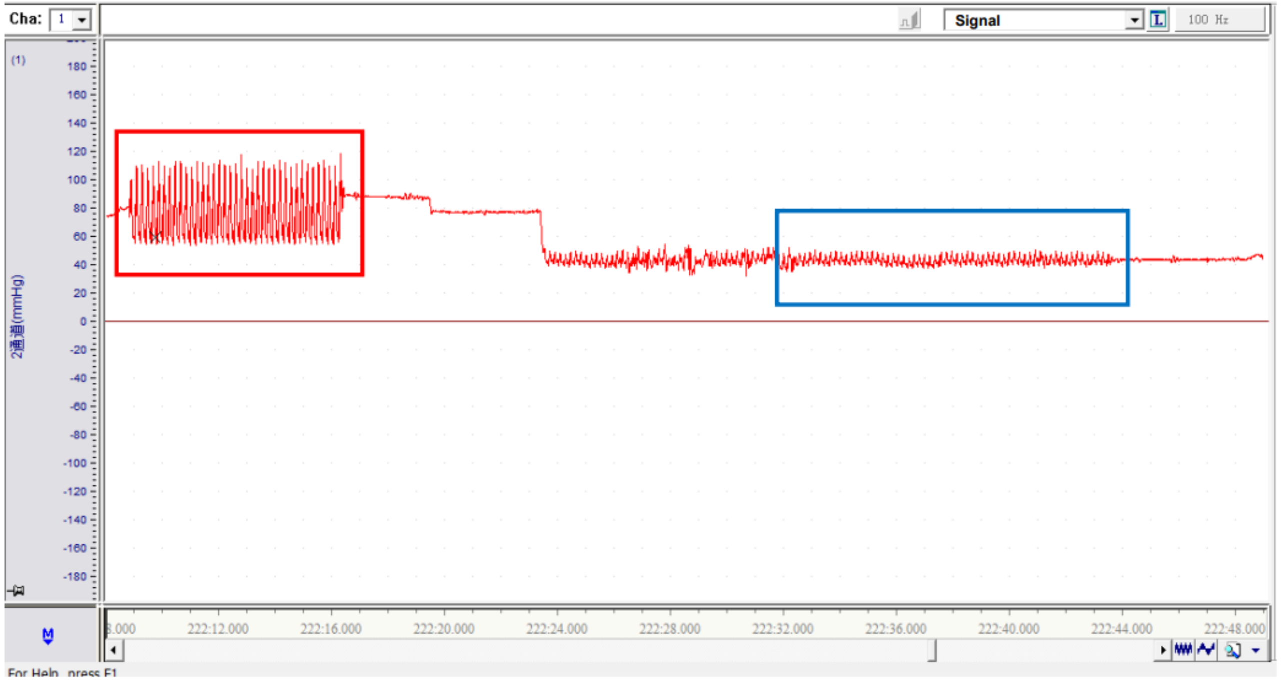

Supplementary Figure 1: Intraoperative arterial pressure measurement diagram. The measurement time point is 10 minutes after VA-ECMO weaning. Red box, septic shock with VA-ECMO treatment. Blue box, septic shock without VA-ECMO treatment; VA-ECMO, venoarterial extracorporeal membrane oxygenation.
