# Supplementary material for: Venoarterial Extracorporeal Membrane Oxygenation Implementation in Septic Shock Rat Model
Source: ASAIO J. 2024 Feb 29;70(8):653–60. doi: 10.1097/MAT.0000000000002168 (PMC11280450; doi:10.1097/MAT.0000000000002168)
Supplement: Supplementary file 3 [file mat-70-0653-s003.pdf]

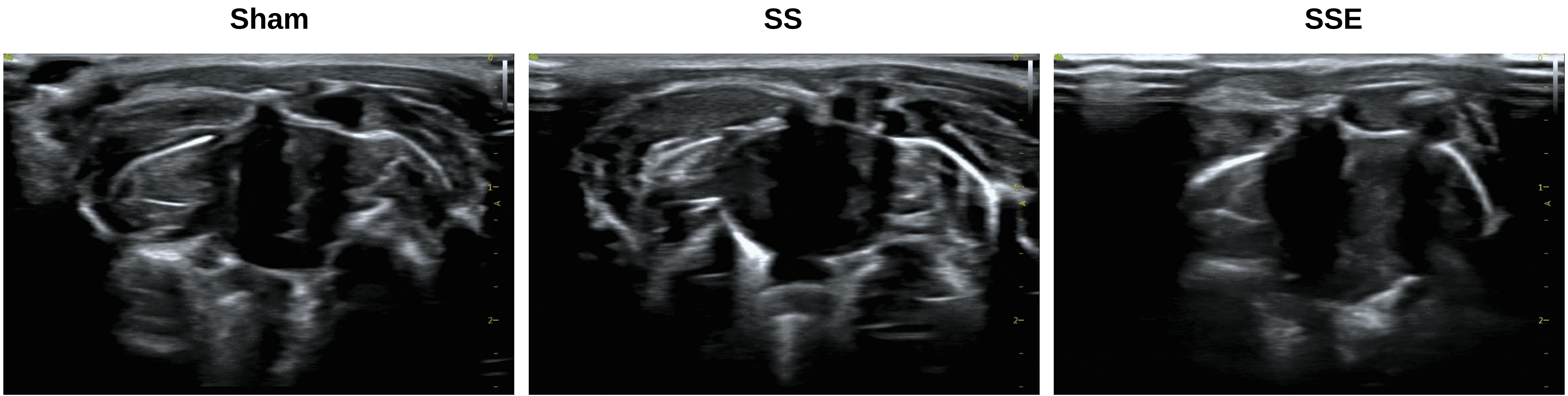

Supplementary Figure 2: Representative of 2-dimensional echocardiography images from an apical 4-chamber view. The septic shock rat exhibited a notable reduction in heart rate and heart muscle contractility. VA-ECMO treatment facilitated the restoration of cardiac function. SS, Septic Shock; SSE, Septic Shock + VA-ECMO; VA-ECMO, venoarterial extracorporeal membrane oxygenation.
